# The green alga *Zygogonium ericetorum* (Zygnematophyceae, Charophyta) shows high iron and aluminium tolerance: protection mechanisms and photosynthetic performance

**DOI:** 10.1093/femsec/fiw103

**Published:** 2016-05-12

**Authors:** Klaus Herburger, Daniel Remias, Andreas Holzinger

**Affiliations:** 1 Institute of Botany, Functional Plant Biology, University of Innsbruck, Sternwartestraße 15, A-6020 Innsbruck, Austria; 2 University of Applied Sciences Upper Austria, School of Engineering, Stelzhamerstraße 23, A-4600 Wels, Austria

**Keywords:** aluminium, cell wall, green algae, iron, photosynthesis, zinc

## Abstract

Streptophyte green algae, ancestors of Embryophytes, occur frequently in terrestrial habitats being exposed to high light intensities, water scarcity and potentially toxic metal cations under acidic conditions. The filamentous *Zygogonium ericetorum* synthesizes a purple vacuolar ferrous pigment, which is lost after aplanospore formation. However, it is unknown whether this cellular reorganization also removes excessive iron from the protoplast and how *Z. ericetorum* copes with high concentrations of aluminium. Here we show that aplanospore formation shifts iron into the extracellular space of the algal filament. Upon germination of aplanospores, aluminium is bound in the parental cell wall. Both processes reduce iron and aluminium in unpigmented filaments. Comparison of the photosynthetic oxygen production in response to light and temperature gradients in two different *Z. ericetorum* strains from an Austrian alpine and a Scottish highland habitat revealed lower values in the latter strain. In contrast, the Scottish strain showed a higher optimum quantum yield of PSII during desiccation stress followed by rehydration. Furthermore, pigmented filaments of both strains exhibited a higher light and temperature dependent oxygen production when compared to the unpigmented phenotype. Our results demonstrate a high metal tolerance of *Z. ericetorum*, which is crucial for surviving in acidic terrestrial habitats.

## Abstract (precis)

The green alga *Zygogonium ericetorum* shows high iron and aluminium tolerance, and these metals are excluded from the cells by special protection mechanisms, allowing high photosynthetic performance in terrestrial habitats.

## INTRODUCTION

Based on their primary adaptation to freshwater, distinct streptophyte green algae belonging to the Zygnematophyceae moved to the limnetic transition zone and further to dry land, which gave rise to the evolution of land plants in the Mid-Ordovician period (Becker and Marin 2009; Wickett *et al.*^2014^). Today, Zygnematophyceae occur worldwide in limnetic, hydro- and aero-terrestrial habitats (e.g. Škaloud 2009; Stancheva, Sheath and Hall 2012; Pichrtová, Hajek and Elster 2014a), representing a gradient of decreasing water availability, increasing light intensity, temperature fluctuation as well as metal toxicity due to their increased abundance in more acidic environments. Investigating green algae's physiological performance and cell morphology in these different habitats allows tracing adaptations, which were necessary for changing the primary limnetic to terrestrial live-style (Herburger and Holzinger 2015). Several studies focused on the photosynthetic performance of Zygnematophyceae under light, temperature, osmotic, water or nutrient starvation stress, while either natural field populations (Holzinger, Roleda and Lütz 2009; Aigner *et al.*^2013^; Pichrtová, Hajek and Elster 2014a), cultured algae (Kaplan *et al.*^2013^; Pichrtová *et al.*^2013^; Pichrtová, Kulichová and Holzinger 2014b; Herburger, Lewis and Holzinger 2015) or both (Hawes 1990; Stamenković and Hanelt 2013) were investigated. Many of these green algae use light very efficiently, indicated by a high photosynthetic performance under dim conditions, low light compensation and saturation points and lacking strong photoinhibition even far beyond light saturation. This high photophysiological plasticity coincides with the hydro- and aero-terrestrial lifestyle, since it was also found in several other streptophyte algae (e.g. Klebsormidiophyceae) from similar habitats (Kaplan *et al.*^2012^; Karsten, Herburger and Holzinger 2014; Herburger, Karsten and Holzinger 2016). Changing light conditions often go along with desiccation stress and cellular water loss strongly suppresses the photosynthetic performance of green algae in the vegetative state (Holzinger and Karsten 2013). It was shown recently that specialized resistant-cells (pre-akinetes or akinetes) formed by *Zygnema* after mild long-term desiccation stress (Pichrtová, Hajek and Elster 2014a) or nutrient starvation (Herburger, Lewis and Holzinger 2015) provide resistance against short-term desiccation stress. However, pre-akinetes exhibit lower maximum photosynthetic rates when compared with vegetative cells (Herburger, Lewis and Holzinger 2015), making the transformation into these resistant cells a trade-off between desiccation tolerance and a high metabolic activity. Another common response of Zygnematophyceae to abiotic stress is the accumulation of phenolic compounds (Remias *et al.*^2011^; Aigner *et al.*^2013^; Pichrtová *et al.*^2013^). In natural habitats, green cells of the filamentous streptophyte green algae *Z. ericetorum* (‘green morph’) accumulate high amounts of phenolics and turn purple (‘purple morph’; Alston 1958). The purple pigment revealed as a glycosylated and highly branched phenolic compound which forms complexes with Fe^3+^ (Newsome and van Breemen 2012), while a ferric (gallate)_2_ complex causes the colour (Newsome, Murphy and van Breemen 2013). Iron is an essential heavy metal trace element for all plants, as it is a major constituent of the cellular redox system including cytochromes, catalase, peroxidase as well as ferredoxin and superoxide dismutase (Karlusich, Lodeyro and Carrillo 2014). On the other hand, due to low pH values, excessive soluble iron (Fe^2+^) can cause oxidative stress by generating reactive oxygen species through the Fenton reaction (e.g. hydroxyl radicals; Cadenas 1989), which are a source of damage to lipids, proteins and nucleic acids (Kranner and Birtić 2005). Therefore, iron metabolism has to be adapted to the cellular demands to maintain a sufficient redox system but to avoid cellular damage because of iron induced oxidative stress (Kampfenkel, Van Montagu and Inzé 1995; Finney and O'Halloran 2003). Important regulators of iron homeostasis in plants are ferritins (Ravet *et al.*^2009^), a superfamily of iron storage proteins (Briat *et al.*^2010^). In the green algae *Chlamydomonas reinhardtii*, ferritins are involved in restoring the PSI level after iron starvation followed by iron repletion and protecting the photosynthetic apparatus under photo-oxidative stress during highlight conditions or iron overflow (Busch *et al.*^2008^). Ferritin-iron complexes of plants are localized in the plastid stroma (Waldo *et al.*^1995^) and mitochondria (Zancani *et al.*^2004^). In contrast, the ferrous phenolic compound responsible for the purple pigmentation in *Z. ericetorum* cells is stored in vacuoles due to its hydrophilic nature (Alston 1958). It has been assumed recently that *Zygogonium* cells can exclude excessive iron from the protoplast into the extracellular space by forming unpigmented aplanospores, while these spores can germinate into new green vegetative cells (Stancheva *et al.*^2014^). Besides iron, low pH values (<5.5), which are typical for habitats of *Z. ericetorum*, can also solubilize other metals into their potentially toxic cationic form (Foy, Chaney and White 1978). Zinc is amongst the most widespread metals in soils, where it is strongly adsorbed until the pH value decreases (Mertens and Smolders 2013), which allows uptake into plant cells (Klimmek *et al.*^2001^). Like iron, zinc is an essential trace metal for plants, but high levels of zinc increase the production of harmful reactive oxygen species (Prasad, Saradhi and Sharmila 1999). Another limiting factor of plant growth is aluminium (Ma, Ryan and Delhaize 2001). In contrast to iron and zinc, aluminium is not essential for plants (Rengel 1992), but its harmful effects occur at even micromolar concentrations (Ma, Ryan and Delhaize 2001). Due to their toxicity, coping with changing and high concentrations of these metal ions is crucial in acidic habitats with strongly fluctuating water availability: Low pH values make metals bioavailable and the evaporation of water can increase the concentration of these ions at even short time scales. However, little information is available on how the ancestors of land plants coped with such stress under natural conditions.

This study investigates cellular and photophysiological responses of *Z. ericetorum* to abiotic stresses prevailing in limnetic transitional habitats, with a focus on resistance against metal toxicity. Therefore, purple *Z. ericetorum* filaments were collected from two different natural field populations in Austria (high alpine habitat: ∼2300 m a.s.l.) and Scotland (forest zone layer: 45 m a.s.l.). The role of cellular reorganization processes (i.e. accumulation of purple pigmentation or formation of aplanospores) to cope with iron, aluminium and zinc stress as well as the photosynthetic response to light, temperature and desiccation gradients was investigated in laboratory experiments. We hypothesize that (i) iron becomes excluded from the protoplasts during aplanospore formation to avoid toxic concentration within the protoplast and that (ii) *Z. ericetorum* is generally well adapted to metal rich environments by either complexing or avoiding the uptake of metals into cells. Furthermore, we tested if (iii) the two *Z. ericetorum* isolates obtained from two different habitats exhibit distinct responses to light, temperature and desiccation gradients, which would imply a high adaptive capacity and phenotypic plasticity of this species. Finally, we investigated whether (iv) the ‘purple morphs’ of both *Z. ericetorum* isolates show higher photosynthetic oxygen productions in response to increasing light and temperature when compared with the respective ‘green morphs’.

## MATERIALS AND METHODS

### Sampling localities, algal origin and experimental setup

Purple *Z. ericetorum* filaments (*Z. ericetorum* AUT ‘purple morph’ = *Zygogonium* AUT-p) were collected in August 2013 on Mt Schönwieskopf (near Obergurgl, Tyrol, Austria). This site was previously sampled by Holzinger, Tschaikner and Remias (2010), Aigner *et al.* (2013) and Stancheva *et al*. (2014, 2016). Purple algal filaments submersed in soil water and original soil water were collected separately in polyethylene vials and transferred immediately to the Institute of Botany at the University of Innsbruck. Physiological measurements and microscopic examinations (see following paragraphs) of *Zygogonium* AUT-p were performed in filtered soil water and started the day after collection. The same day, subsamples were washed, transferred to 100 mL Bold's Basal Medium (BBM, pH = 6.8; Bischoff and Bold 1963) in 250 mL Erlenmeyer flasks. They were cultured in a dark/light regime of 16:8 h in a Percival PGC 6L thermostat (Percival Scientific, Perry, GA, USA) at 20°C and ∼33 μmol photons m^−2^ s^−1^ (adjusted with Osram Daylight Lumilux Cool White lamps L36W/840; Osram. Munich, Germany). In the dark period, temperature was reduced to 14.5°C. Algal filaments turned green (i.e. *Z. ericetorum* AUT ‘green morph’ = *Zygogonium* AUT-g) within 10–12 days. All physiological measurements and microscopic examinations of *Zygogonium* AUT-g started two weeks after subsampling. A second purple sample of *Z. ericetorum* (*Z. ericetorum* SCOT ‘purple morph’ = *Zygogonium* SCOT-p) was collected in August 2014 near Glencoe (Lochaber, Scotland; coordinates in Table 1). Filaments were stored in original soil water obtained from the centre of the population. They were transferred to the laboratory in Innsbruck within one day and treated the same like the samples from Austria (see above) to establish *Z. ericetorum* SCOT ‘green morph’ (= *Zygogonium* SCOT-g) within ∼12 days and perform physiological and microscopic experiments. The methods applied to *Zygogonium* AUT-p, *Zygogonium* AUT-g, *Zygogonium* SCOT-p and *Zygogonium* SCOT-g are summarized in Fig. S1 (Supporting Information).

### Iron and aluminium content in soil water

The iron and aluminium content of the soil water of *Zygogonium* AUT-p and *Zygogonium* SCOT-p were quantified colorimetrically by a Merck Spectroquant iron or aluminium test kit (1.14761.0001 (Fe), 1.14825.0001 (Al); concentration range 0.005–5 mg Fe L^−1^ or 0.02–1.2 mg Al L^−1^) according to the manufacturer's protocol (Merck, Darmstadt, Germany). For sample preservation prior analyses, 1 mL of nitric acid (65%; Sigma-Aldrich, Steinheim, Germany) per litre soil water was added to the soil water. A calibration curve was established by FeCl_2_·4 H_2_O or AlCl_3_ (both Sigma-Aldrich) and the metal content in the water expressed per mg L^−1^. The pH value of the soil water was measured in an untreated sample. Additionally, soil water of three other habitats in Obergurgl (Tyrol, Austria) located at least 500 m away from the habitat of *Zygogonium* AUT-p and predominated by *Z. ericetorum* (‘green morph’, field sample), *Zygnema* sp./ *Z. ericetourm* (‘green morph’, field sample) or *Spirogyra* sp. was analysed as described above for comparison. Iron and aluminium quantification were performed in four independent replicates (*n* = 4).

### Metal quantification in *Zygogonium* AUT

Trace metal concentration (soluble Fe, Al and Zn) of *Zygogonium* AUT-p or *Zygogonium* AUT-g was determined by ICP OES (inductively coupled plasma/optical emission spectrometer; ULTIMA 2 Horiba Jobin Yvonne, Bensheim, Germany) equipped with an AS 80 auto sampler using the software ICP Analyst 5.2. The argon plasma torch (Air Liquide, >99 999%) was generated with a power of 1000 Watt and a flow rate of 12 L min^−1^. Dried algal samples were grinded with a mortar and extracted with methyl tert-butyl ether. Then, a phase separation against 20% ethanol was performed and both phases were evaporated and transferred to water and centrifuged at 13 000 *g* prior use. The clear supernatant was diluted ten times with nitric acid (67%–70%, c = 0.1 mmol L^−1^; Fisher Scientific, Vienna, Austria) in a 50 mL plastic screw tube. Samples were injected by a concentric Meinhard nebulizer in the concentric cyclone chamber. As calibration, the method of external standard was applied between 100 and 10.000 μg L^−1^. A multi standard was used (ICP-Standard-Solution 28 el. Roti Star, Carl Roth, Karlsruhe, Germany).

### Haematoxylin staining and light microscopy

Haematoxylin was used frequently to stain iron and aluminium, which can be discriminated by colouration (Avwioro 2011; Fig. S2, Supporting Information). For haematoxylin staining, filaments of *Zygogonium* AUT-p and Zygogonium AUT-g were washed with A. dest. (3 × 10 min) followed by incubation in a 0.2% haematoxylin solution (Polle, Konzak and Kattrick 1978; Hollborn and Söhne, Leipzig, Germany) for 40 min at room temperature (RT) and washing (3 × 10 min in A. dest.). Stained and unstained (control) filaments were investigated with a Zeiss (Carl Zeiss AG, Jena, Germany) Axiovert 200M microscope (63 × 1.4 NA objective). Optical contrast was enhanced by using differential interference contrast (DIC). Images were captured with an Axiocam MRc5 camera using Zeiss Axiovision software. All images were further processed with the software Adobe Photoshop (CS5) version 12.1 (Adobe Systems, San José, CA, USA).

### Morin Staining and confocal laser scanning microscopy

Morin (2',3,4',5,7-pentahydroxyflavone; VWR, Darmstadt, Germany) forms highly specific complexes with aluminium at pH = 5 (Browne, McColl and Driscoll 1990). Filaments of *Zygogonium* AUT–p and *Zygogonium* AUT-g were stained according to Navascués *et al.* (2012) with modifications: Filaments were rinsed in A. dest., washed in buffer (5 mM ammonium acetate (Sigma-Aldrich), pH = 5, 3 × 10 min), stained in morin (100 μM in buffer, 10 min) and washed in buffer (2 × 10 min). A Zeiss Pascal 5 CLSM system equipped with an argon laser (excitation 488 nm) on a Zeiss Axiovert 200 M was used to visualize morin (emission 505–550 nm, false colour green) and the chloroplast autofluorescence (emission 560 nm long pass filter, false colour red). A corresponding bright field image was merged with the autofluorescence image.

### Transmission electron microscopy

Fixation for transmission electron microscopy (TEM) followed the protocol of Holzinger, Tschaikner and Remias (2010). Briefly, filaments of *Zygogonium* SCOT-p were fixed in sodium cacodylate puffer (10 mM, pH 6.8) containing 1.25% glutaraldehyde for 1.5 h. Filaments were transferred to 1% OsO_4_ and postfixed at 4°C for 16 h. After dehydration in increasing ethanol concentrations and propylene oxide, filaments were embedded in modified Spurr's resin, sectioned on a Leica ultracut (Leica Microsystems, Wetzlar, Germany), counterstained (uranyl acetate, Reynold's lead citrate) and examined with a Zeiss LIBRA 120 TEM at 80 kV connected to a Proscan 2 k SSCCD camera (Proscan Electronic Systems, Lagerlechfeld, Germany).

### Photosynthetic oxygen measurements—light and temperature dependence

Photosynthesis irradiance (PI) curves were recorded according to Remias, Albert and Lütz (2010). Briefly, 3 mL of algal suspension (purple or green morph of *Zygogonium* AUT or *Zygogonium* SCOT) enriched with 2 mM NaHCO_3_ (final concentration) were filled in a thermostatic acrylic chamber on a magnetic stirrer and connected to a Presens Fibox 3 oxygen optode (Presens, Regensburg, Germany). Algae were exposed to eight light intensities (3–500 μmol photons m^−2^ s^−1^) at 20°C for always 6 min. In the dark period (6 min) directly before and after light measurements, respiratory oxygen consumption (R) was recorded and averaged to express R. After each measurement, chlorophyll (chl.) *a* was quantified according to Porra, Thompson and Kriedmann (1989) to estimate μmol O_2_ h^−1^ mg^−1^ chl. *a* in response to increasing light intensities as described by Herburger, Lewis and Holzinger (2015). PI curves were fitted by different models depending on whether photoinhibition occurred (Walsby 1997) or not (Webb, Newton and Starr 1974) to derive α, positive slope at limiting photon fluence rates (μmol O_2_ h^−1^ mg^−1^ chl. *a* (μmol photons^−1^ m^−2^ s^−1^)^−1^); I_c_, light compensation point (μmol photons m^−2^ s^−1^); I_k_, initial value of light-saturated photosynthesis (μmol photons m^−2^ s^−1^); and P_max_, maximum photosynthetic oxygen production under light saturation (μmol O_2_ h^−1^ mg^−1^ chl. *a*). To compare the PI curve kinetics of the purple or green morphs of *Zygogonium* AUT to *Zygogonium* SCOT and the purple to the green morph of *Zygogonium* AUT or *Zygogonium* SCOT to each other, linear regressions between the mean values of O_2_ production (μmol O_2_ h^−1^ mg^−1^ chl. *a*) at PAR 0, 3, 10, 14, 30, 70, 125, 290 and 500 μmol photons m^−2^ s^−1^ were calculated, and R^2^ was derived. Temperature dependence of photosynthetic oxygen production and respiratory consumption were examined according to Herburger, Lewis and Holzinger (2015). Three mL of algal suspension (purple or green morph of *Zygogonium* AUT or *Zygogonium* SCOT), enriched with 2 mM NaHCO_3_, were exposed to a temperature gradient (5°C–45°C in nine Δ5°C steps) at 100 μmol photons m^−2^ s^−1^. O_2_ production (gross photosynthesis) and consumption (respiration) were expressed in μmol O_2_ h^−1^ mg^−1^ chl. *a*, and net photosynthesis and gross photosynthesis:respiration (P/R) ratios were calculated for each temperature step.

### Maximum quantum yield of PSII (Fv/Fm)—desiccation and rehydration response

Reduction and recovery of the maximum quantum yield of PSII (Fv/Fm) in response to desiccation followed by rehydration in *Zygogonium* SCOT-p and *Zygogonium* SCOT-g were measured according to Aigner *et al.* (2013). Algal filaments were transferred to Whatman (Whatman, Dassel, Germany) GF/F glass fibre filters (*n* = 6) in a desiccation chamber (Karsten, Herburger and Holzinger 2014) and dried at ambient air humidity and constant light (∼30 μmol photons m^−2^ s^−1^) and temperature (21 ± 0.5°C). A pulse-amplitude modulated fluorimeter (PAM 2500; Heinz Walz GmbH, Effeltrich, Germany) placed outside the chamber was used to measure the F_v_/F_m_ value during desiccation and subsequent rehydration with filtered soil water (*Zygogonium* SCOT-p) or culture medium (*Zygogonium* SCOT-g).

### Microscopic imaging PAM

The effective quantum yield of PSII (Y(II); false coloured image) and near-infrared remission (NIR, 780 nm) were visualized at the subcellular level with the microscopic version of an Imaging-PAM (M-series, Heinz Walz GmbH, Herburger and Holzinger 2015). Therefore, algal filaments of *Zygogonium* AUT-p and *Zygogonium* AUT-g exhibiting different cell morphologies (younger or older filaments, different stages of aplanospore formation and pigmentation) were collected by using a stereomicroscope (Lumar V12; Carl Zeiss AG) and transferred to welled slides. A modified Axio Scope A.1 epifluorescence microscope equipped with a Zeiss Fluar 40 × 1.3 NA objective and CCD Camera IMAG-K6 controlled with ImagingWinGigE (V2.45i) software was used for image generation. Measuring light for Y(II) determination was provided by a LED (620 nm). The signal/noise ratio was not modified by a SP-Routine.

### Statistical evaluation of the data

Comparisons of soil water's iron and aluminium contents of five different habitats (*n* = 4), photosynthetic parameters derived from PI curves (α, I_c_, I_k_, P_max_, R; *n* = 3), effects of temperature (O_2_ production and consumption, P:R ratios; *n* = 3) or desiccation (F_v_/F_m_; *n* = 6) on photosynthesis were performed by one-way analysis of variance (ANOVA) followed by Tukey's post hoc test (*P* < 0.05) to find homogeneous subgroups of significantly different means. Analyses were carried out in Origin 8.5 software (OriginLab Corporation, Northampton, MA, USA).

## RESULTS

### Habitat characteristics

*Zygogonium* AUT-p filaments formed dense purple layers submersed into a shallow puddle (depth: ∼5–20 cm), which was drained by a streamlet (Fig. S3A, Supporting Information). Occasionally, aggregates of green filaments were found in deeper layers of the puddle and covered by the ‘purple morph’. Compared to the surrounding, vascular plants were less abundant inside the drained population of *Zygogonium* AUT-p (Fig. S3A, Supporting Information). On the sides with lower water availability (i.e. outside the pond and streamlet), *Zygogonium* AUT-p was scarce. The Scottish site was dryer compared to the habitat in Obergurgl (Fig. S3B, Supporting Information), since it was not drained by a streamlet and rainwater was the only water source. In this habitat, a thin layer of dark purple *Zygogonium* SCOT-p filaments coated by a thin water film covered the gravelly soil (Fig. S3C, Supporting Information), while green filaments (<5%) or co-occurring vascular plants were scarce (Fig. S3B, Supporting Information). Occasionally, filamentous cyanobacteria were observed in both habitats. Detailed habitat characteristic including metrological data are listed in Table 1.

**Table 1.** Origin of *Zygogonium* AUT-p and *Zygogonium* SCOT-p.

Isolate	Habitat	Metrological data
*Zygogonium ericetorum* AUT ‘purple morph’ (*Zygogonium* AUT-p)	Streamlet on Mt Schönwieskopf (∼2300 m a.s.l.), Tyrol, Austria (46.84804°N 11.01539°E); isolated on 18 August 2013; water depth 5–20 cm, open water surface; air temperature: 8.5°C	Minimum air temperature: −8°C to –6°C Maximum air temperature: −3°C to 12°C Monthly rainfall days: 11–18 Monthly precipitation: 36.3–120.3 mm Annual rainfall: 839 mm
*Z. ericetorum* SCOT ‘purple morph’ (*Zygogonium* SCOT-p)	Wayside near Glencoe (∼45 m a.s.l.), Lochaber, Scotland (56.684891°N −5.090494°E); isolated on 16 Aug 2014; algal filaments covered by thin water layer; air temperature: 16.9°C	Min. air temperature: −0.7°C to 9.7°C Max. air temperature: 5.5°C–17.4°C Monthly rainfall days: 14–20 Monthly precipitation: 77.0–248.3 mm Annual rainfall: 1809.4 mm

Habitat characteristics and meteorological data (temperatures expressed as means, www.worldweatheronline.com, http://www.metoffice.gov.uk ) are given.

### Iron and aluminium content in soil water

**Figure 1.**
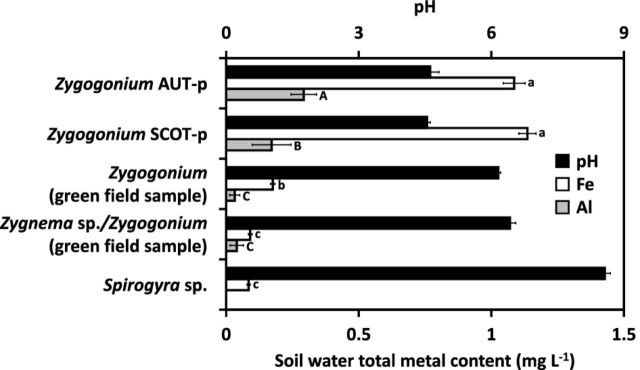
Comparison of the photometrically detected iron and aluminium content (*n* = 4) and pH value of soil water from *Zygogonium* AUT-p, *Zygogonium* SCOT-p and three different Austrian habitats containing a ‘green morph’ of *Zygogonium*, a mixture of *Zygnema* sp. and a ‘green morph’ of *Zygogonium* or *Spirogyra* sp. (*n* = 4 ± SD). Significant differences between the groups are indicated by lower case (iron content) or capital (aluminium content) letters. Data were analysed by one-way ANOVA followed by Tukey's post hoc test (*P* < 0.05).

The iron and aluminium content between the soil water of habitats of *Zygogonium* AUT-p, *Zygogonium* SCOT-p and the soil water of three other habitats predominated by zygnematalean green algae in Obergurgl (Austria) differed significantly (Fig. 1). The highest iron contents were measured in the habitats of *Zygogonium* AUT-p (1.09 mg Fe L^−1^) and *Zygogonium* SCOT-p (1.14 mg Fe L^−1^; Fig. 1). Habitats predominated by a ‘green morph’ of *Z. ericetorum* (0.18 mg Fe L^−1^), a ‘green morph’ of *Z. ericetorum* and *Zygnema* sp. (0.09 mg Fe L^−1^) or *Spirogyra* sp. (0.08 mg Fe L^−1^) contained significantly less iron (Fig. 1). The highest aluminium content occurred in the habitat of *Zygogonium* AUT-p (0.29 mg Al L^−1^; Fig. 1). Significantly, lower values occurred in the site of *Zygogonium* SCOT-p (0.17 mg Al L^−1^; Fig. 1). The aluminium content was very low in habitats predominated by *Zygnema* sp. and a ‘green morph’ of *Z. ericetorum* and not measurable in the habitat of *Spirogyra* sp. due to too low concentrations (Fig. 1). The pH values in the soil water samples of *Zygogonium* AUT-p and *Zygogonium* SCOT-p (∼4.6) were lower when compared with the three other sites (∼6.2–8.6; Fig. 1).

### Metal quantification in *Zygogonium* AUT

Filaments of *Zygogonium* AUT-p contained much more Al, Zn and Fe when compared to *Zygogonium* AUT-g, where aluminium showed higher values compared to zinc and iron (Table 2).

**Table 2.** Mean (*n* = 2) and range (parentheses) of metal concentrations (μg mg dry mass^−1^) in *Zygogonium* AUT-p and *Zygogonium* AUT-g measured by ICP OES.

	Al	Zn	Fe
*Zygogonium* AUT-p	**0.938**	**0.844**	**0.459**
	(0.683–1.193)	(0.567–1.121)	(0.187–0.730)
*Zygogonium* AUT-g	**0.248**	**0.003**	**0.054**
	(0.194–0.303)	(0.002–0.003)	(0.042–0.066)

### Light microscopy

**Figure 2.**
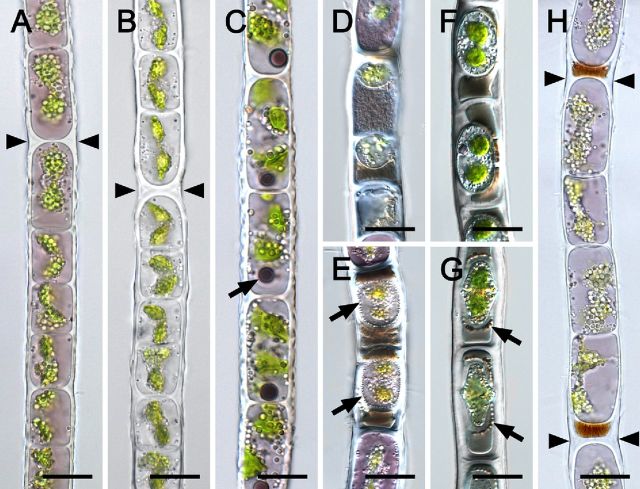
Light micrographs of vegetative cells (**A**–**C**, **F**–**H**) and aplanospores (**D**, **E**) of *Zygogonium* SCOT (A, C–H: *Zygogonium* SCOT-p, B: *Zygogonium* SCOT-g). (A), (B) H-shaped cell wall structures (arrowheads). (C) One spherical dark purple inclusion per cell (arrow), dentate longitudinal cell walls. (D) Small aplanospores; brownish or purple granular cytoplasmic residue outside the spores. (E) Aplanospores embedded in brownish cytoplasmic residue and; numerous transparent particles in the periphery of the spore (arrows). (F) Encysted cells with two prominent chloroplasts. (G) Encysted cells surrounded by ‘highly ordered’ spherical bodies (arrows). (H) Purple filament formed by germination of aplanospores as indicated by the brownish remnant of cytoplasmic residue; H-shaped cell wall structures (arrowheads). Bars = 20 μm.

Cell morphology and ultrastructure of vegetative and conjugating filaments of *Zygogonium* AUT-p have been described in detail previously (Holzinger, Tschaikner and Remias 2010; Aigner *et al.*^2013^; Stancheva *et al.*^2014^, ^2016^). Thus, light microscopic examination in this study focused on *Zygogonium* SCOT-p and *Zygogonium* SCOT-g, which also exhibited vegetative cells with a high morphological variability (Fig. 2). Around >95% of the filaments obtained from the natural population were purple with one centrally located nucleus, two plate-like shaped chloroplasts surrounded by transparent particles, which were scarce in the cell periphery (Fig. 2A). Rarely (∼2%), filaments exhibited spherical dark purple inclusions near one cell pole and dentate outer cell walls (Fig. 2C). After two weeks of cultivation in BBM, the purple pigmentation was lost (Fig. 2B), while ∼25% of the filaments stored in soil water from the habitat (pH = ∼4.6) remained purple for several weeks. H-shaped cell wall structures were observed frequently in both the purple and green morph (Fig. 2A and B). Aplanospores occurred frequently and appeared similar as described in detail by Stancheva *et al.* (2014), including the spores being attached to the cross cell walls of the vegetative cell and dark cytoplasmic residue outside the spores (Fig. 2D and E). Occasionally (∼1% of filaments), a type of cell encystment was observed, where encysted cells occupied the centre of vegetative cells (Fig. 2F and G). These encysted cells contained ‘highly ordered’ transparent particles in the periphery of their lumen, and they were embedded in dark cytoplasmic residue (Fig. 2F and G). Encysted cells lacked highly condensed cytoplasm and cell organelles common in aplanospores. Germination of encysted cells was not observed. In contrast, aplanospores germinated into new vegetative cells, pushing the cytoplasmic residue outside the cells to H-shaped cell wall structures (Fig. 2H), where fragmentation took place frequently (not shown). This process occurred in natural field samples (*Zygogonium* AUT-p) and in subsamples after transferring them to BBM. In contrast, aplanospore formation and germination was not observed in Austrian habitats predominated by the ‘green morphs’ of *Z. ericetorum*.

### Haematoxylin staining

**Figure 3.**
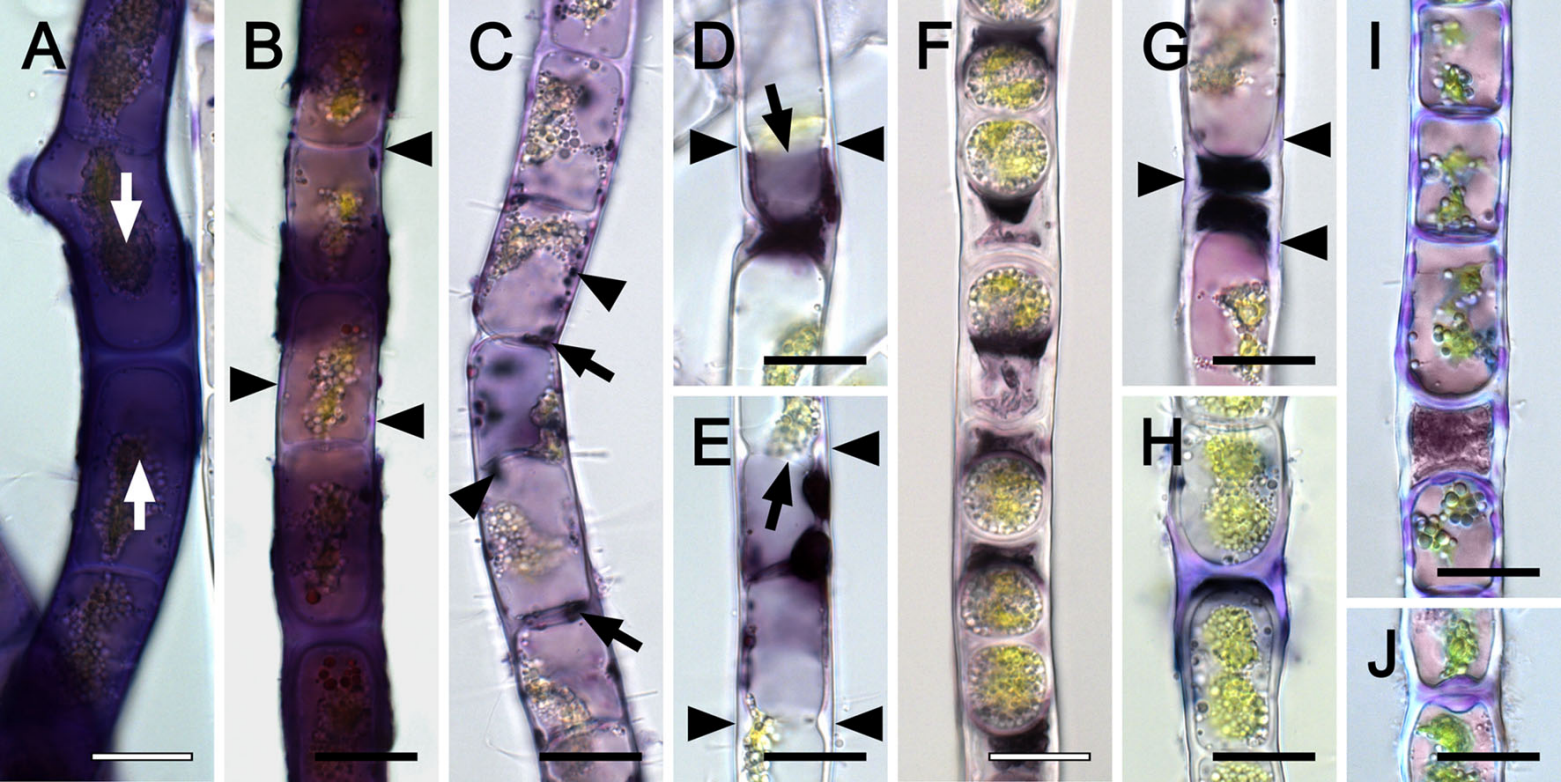
Haematoxylin staining of *Zygogonium* AUT-p (**A**–**G**) and *Zygogonium* AUT-g (**H**–**J**) filaments. (A) Strong violet staining in the cell and in H-shaped cell wall structure (arrows). (B) Lilac (arrowheads) and dark violet to blackish staining in the cell wall. (C) Dark violet to blackish staining inside the protoplast (arrowheads) and cross cell walls (arrows). (D, E) Strong and sharply delineated staining in the protoplast near cell poles (arrow); deposition of cell wall material (arrowheads). (F) Strong blackish staining in the extracellular space outside unpigmented aplanospores. (G) Germinated aplasnpores with lilac staining in the cell wall (arrowheads). (H) Lilac staining in H-shaped cell wall structure, (I) longitudinal and cross cell walls and (J) cross cell wall protuberances. Bars = 20 μm.

Incubating *Zygogonium* AUT-p in haematoxylin stained the cell walls of ∼10% of the filaments entirely dark violet to blackish, while particularly strongly stained sheaths covered H-shaped cell wall structures between individual cells (Fig. 3A). Frequently, dark violet to blackish staining restricted to H-shaped cell wall structures occurred and lilac stained areas of the cell walls were found between these structures (Fig. 3B). In contrast, filaments lacking strong staining in or on the outer cell walls, showed dark violet to blackish staining of spherical bodies in the protoplast with a maximum in the cell periphery and cross cell walls (Fig. 3C). In filaments depositing cell wall material close to the cross cell walls (i.e. beginning aplanospore formation), blackish staining was restricted to chloroplast-free areas (Fig. 3D and E). Aplanospores lacked staining, while it was particularly strong in the cytoplasmic residue outside the spores (Fig. 3F). Germinated aplanospores, which were either purple (Fig. 3G) or green (Fig. 3H), lacked staining, which was restricted to delimited areas adjacent to the spores. Occasionally, green filaments showed lilac haematoxylin staining in specific areas of the outer and cross cell walls (Fig. 3I), while cross-wall protuberances (Fig. 3J) and H-shaped cell wall structures were stained frequently (Fig. 3H).

### Morin staining

**Figure 4.**
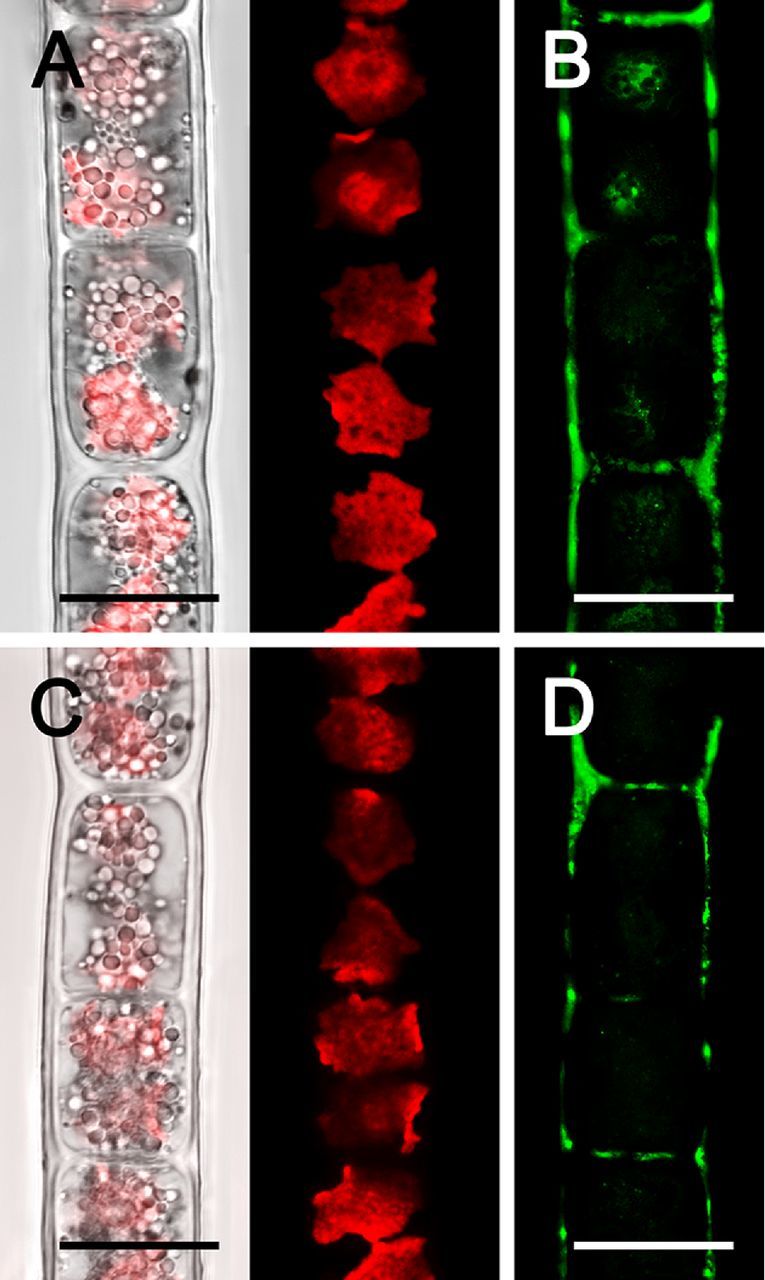
Confocal laser scanning micrographs (**A**, **C**) and morin (green) staining (**B**, **D**) of *Zygogonium* AUT-p (A, B) and *Zygogonium* AUT-g (C, D). (A, C) Bright field image merged with corresponding chloroplast autofluorescence (red) image. Bars = 20 μm.

Aluminium was detected by morin staining in both *Zygogonium* AUT-p and *Zygogonium* AUT-g, with a maximum in H-shaped cell wall structures (Fig. 4). *Zygogonium* AUT-p showed stronger labelling in the cell walls and inside one third of the cells in the pyrenoid regions (Fig. 4B). In contrast, *Zygogonium* AUT-g lacked staining inside cells (Fig. 4D). Visualization of the chloroplast autofluorescence by confocal laser scanning microscopy (CLSM) revealed variably shaped chloroplasts in vegetative cells, ranging from a rounded to irregularly rounded appearance with several protrusions in one plane (Fig. 4A and C). Controls, where morin was omitted, lacked fluorescence in the green channel (not shown).

### Transmission electron microscopy

**Figure 5.**
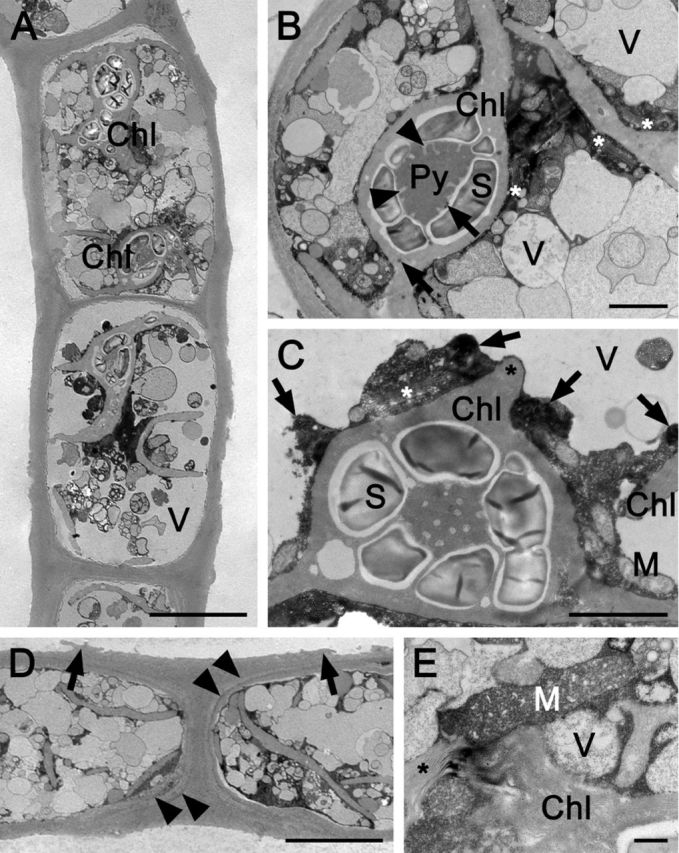
Transmission electron micrographs of longitudinal sections through *Zygogonium* SCOT-p. (**A**) Overview of two cells. (**B**) Detail of the protoplast: one pyrenoid with tubular invaginations, starch grains; plastoglobules (arrows); numerous vacuoles; Golgi bodies (asterisks). (**C**) Detail of a chloroplast with protruding wing (asterisk), pyrenoid, starch grains, mitochondria, Golgi bodies (asterisk), electron-dense structures (arrows) and vacuoles. (**D**) H-shaped cell wall structure; cell wall layers outside the innermost layer surrounding the cells are marked with arrowheads; H-shaped structures detach toward their edges (arrows). (**E**) Enlarged mitochondrion with distinctly bloated cristae; Golgi bodies (asterisk). Chl chloroplast, Py pyrenoid, M mitochondrion, S starch grain and V vacuole. Bars = 10 μm (a, d), 2 μm (b, c) and 0.5 μm (e).

Each cell of *Zygogonium* SCOT-p contained two plate-like chloroplasts with an enlarged central part, holding one pyrenoid, which was invaginated by thylakoid membranes and surrounded by one layer of large starch grains (Fig. 5A–C). Occasionally, additional starch grains were deposited throughout the chloroplast (Fig. 5A). Plastoglobules were scarce (Fig. 5B). Sometimes, thylakoid membranes containing wings protruded from the central part of the chloroplast (Fig. 5C). Cells were filled with numerous vacuoles with different sizes (diameter 0.1–9 μm) and appearances (electron—opaque or granulated contents; Fig. 5A and B). Several Golgi bodies were located closely to the chloroplasts (Fig. 5B). Longitudinal and cross cell walls were layered and had a diameter of 0.6–3.5 μm (Fig. 5B and D). Prominent layered H-shaped cell wall structures between individual cells were observed frequently, which ruptured towards their edges (Fig. 5D). Occasionally, mitochondria were distinctly enlarged and cristae bloated (Fig. 5E).

### Light dependence of photosynthesis

**Figure 6.**
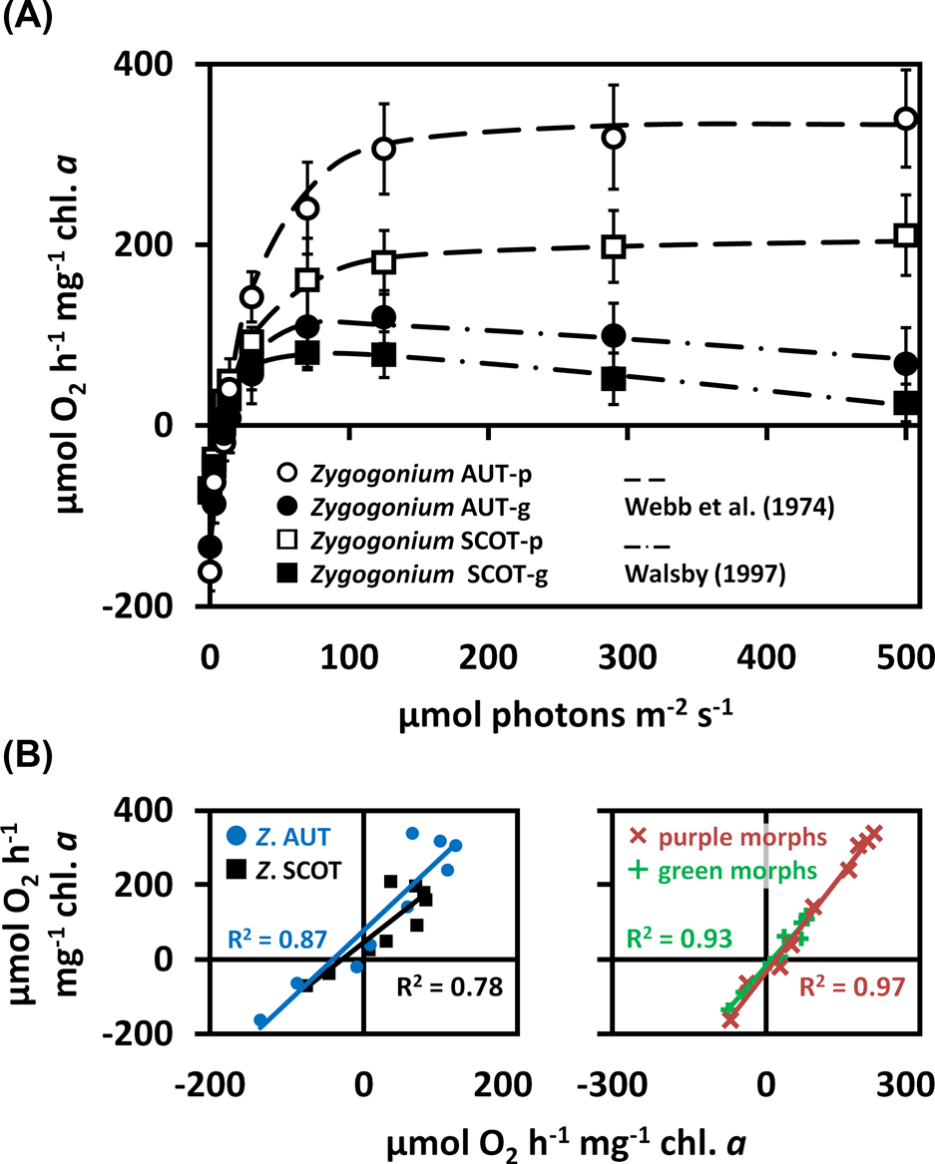
(**A**) Photosynthetic O_2_ production and respiratory consumption as a function of increasing PAR up to 500 μmol photons m^−2^ s^−1^ measured in the purple and green morphs of *Zygogonium* AUT and *Zygogonium* SCOT (*n* = 3 ± SD). Data points of the ‘purple morphs’ and ‘green morphs’ were fitted according to Webb, Newton and Starr (1974) and Walsby (1997), respectively. (**B**) Linearly correlated O_2_ production/consumption at increasing PAR. Right: correlations of the ‘purple morph’ (*x*-axis) with the ‘green morph’ (*y-*axis) of *Zygogonium* AUT or *Zygogonium* SCOT. Left: correlations of the two ‘purple morphs’ or ‘green morphs’ of *Zygogonium* AUT (*y-*axis) and *Zygogonium* SCOT (*x-*axis) to each other. Coefficients of determination (R^2^) are shown.

Photosynthetic oxygen production and respiratory consumption in response to light gradients depended on the habitats the algae were obtained from and whether pigmentation was present or not (Fig. 6A). *Zygogonium* AUT-p showed the highest maximum O_2_ production (P_max_) and respiratory consumption (R), followed by *Zygogonium* SCOT-p (Fig. 6A; Table 3). In general, both ‘green morphs’ exhibited lower P_max_ and R values when compared to the ‘purple morphs’, while the lowest values were measured in *Zygogonium* SCOT-g (Fig. 6A; Table 3). In contrast to *Zygogonium* AUT, R did not differ significantly between the green and purple morph of *Zygogonium* SCOT (Table 3). *Zygogonium* AUT exhibited significantly (*P* < 0.05) higher α and I_c_ values compared to *Zygogonium* SCOT, while the two morphs of the same isolate did not differ significantly (*P* < 0.05) from each other (Table 3). In contrast, the I_k_ values of both ‘purple morphs’ were similar but significantly (*P* < 0.05) lower in the ‘green morphs’, while *Zygogonium* SCOT-g exhibited the lowest value (Table 3). Linear regression between the photosynthetic oxygen production and respiratory consumption from PAR 0 to 500 μmol photons m^−2^ s^−1^ in the purple and green morph of *Zygogonium* AUT and *Zygogonium* SCOT revealed a strong linear correlation (Fig. 6B). Similarly, the light curve data of the two different ‘purple morphs’ or ‘green morphs’, respectively, were strongly linearly correlated to each other (Fig. 6B).

**Table 3.** Photosynthetic parameters of PI curves (O_2_) of the purple and green morph of *Zygogonium* AUT and *Zygogonium* SCOT, calculated by using the fitting models of Walsby 1997 (‘green morphs’) or Webb, Newton and Starr 1974 (‘purple morphs’).

	α	I_c_	I_k_	P_max_	R
*Zygogonium* AUT-p	11 ± 2.64^a^	12.99 ± 2.02^a^	28.42 ± 5.23^a^	315.5 ± 28.38^a^	−161.93 ± 15.3^a^
*Zygogonium* AUT-g	13.12 ± 2.17^a^	11.77 ± 0.95^a^	16.76 ± 2.99^b^	113.86 ± 14.79^b^	−134.57 ± 12.72^b^
*Zygogonium* SCOT-p	7.89 ± 1.27^b^	7.5 ± 1.27^b^	29.79 ± 8.01^a^	148.95 ± 18.08^c^	−69.48 ± 6.64^c^
*Zygogonium* SCOT-g	9.23 ± 1.83^b^	8.08 ± 0.95^b^	11.17 ± 1.12^b^	103.81 ± 12.89^b^	−74.36 ± 11.64^c^

Different lower case letters indicate significant differences between the values (α, I_c_, I_k_, P_max_ or R) of the four different groups. They were determined by one-way ANOVA followed by Tukey's post hoc test (*P* < 0.05). α initial slope in the light-limiting range (μmol O_2_ h^−1^ mg^−1^ chl. a (μmol photons m^−2^ s^−1^)-1), I_c_ light compensation point (μmol photons m^−2^ s^−1^), I_k_ initial value of light-saturated photosynthesis (μmol photons m^−2^ s^−1^), P_max_ maximum photosynthetic rate in the light-saturated range (μmol O_2_ h^−1^ mg^−1^ chl. a), R respiration rate in the dark (μmol O_2_ h^−1^ mg^−1^ chl. a).

### Temperature dependence of photosynthesis

**Figure 7.**
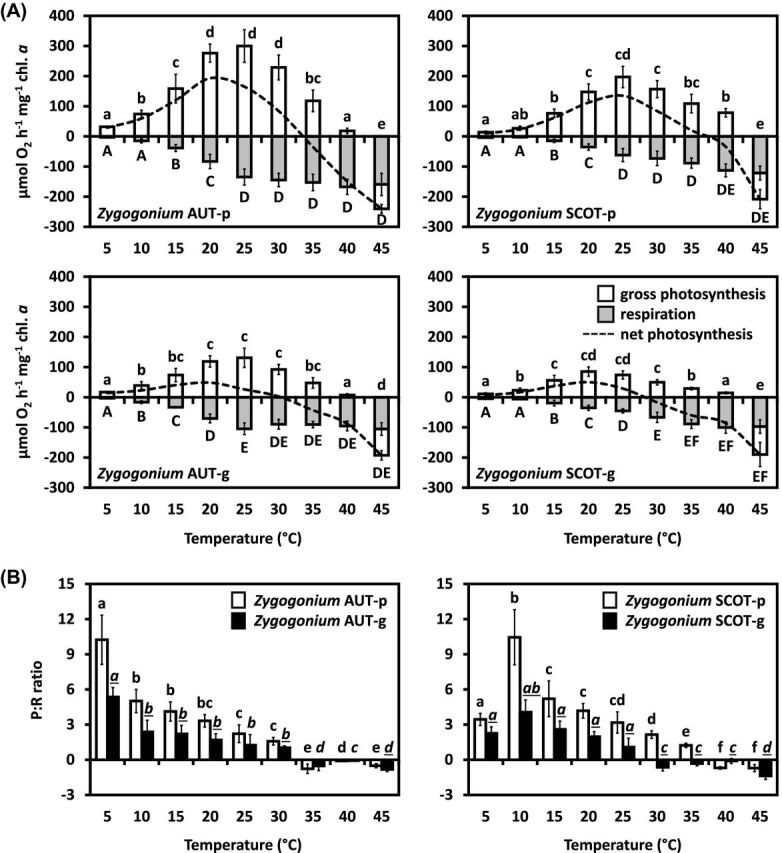
Temperature dependence of photosynthesis in the ‘purple morph’ and ‘green morph’ of *Zygogonium* AUT and SCOT. (**A**) Gross and net photosynthetic O_2_ production and respiratory consumption in response to increasing temperatures (0°C –45°C; *n* = 3 ± SD; SD values for net photosynthesis are excluded to increase legibility). (**B**) Gross photosynthesis: respiration (P:R) ratios. Significant temperature depended differences (lower case letters: gross photosynthesis, capital letters: respiration, lower case and cursive letters: P:R ratios) were determined by one-way ANOVA, followed by Tukey's post hoc test (*P* < 0.05). Significant different P:R ratios of the ‘purple morph’ and the ‘green morph’ of the same strain at the same temperature steps (underlined letters) were determined by standard two-sample *t*-tests (*P* < 0.05).

In all four samples investigated (*Zygogonium* AUT-p/g and SCOT-p/g), photosynthetic oxygen production and respiratory consumption were strongly temperature-dependent (Fig. 7A). Both *Zygogonium* AUT-p and *Zygogonium* SCOT-p exhibited a linearly increasing O_2_ production from 5°C to 20°C (AUT) or to 25°C (SCOT), followed by a decrease to negative values at 45°C. At 40°C, positive oxygen production was still measurable, while *Zygogonium* SCOT-p showed a higher O_2_ production at this temperature step when compared with *Zygogonium* AUT-p (Fig. 7A). Respiratory oxygen consumption increased linearly from 5°C and reached a broad temperature maximum between 25°C and 45°C (AUT) or 40°C and 45°C (SCOT; Fig. 7A). In general, both *Zygogonium* AUT-g and *Zygogonium* SCOT-g exhibited a lower photosynthetic oxygen production in response to increasing temperatures from 5°C to 45°C (Fig. 7A). Gross photosynthetic O_2_ production increased linearly from 5°C to 20°C (AUT) or 5°C to 20°C (SCOT), followed by a linearly decrease and reaching negative values at 45°C in the ‘green morphs’ (Fig. 7A).The lower temperature depended photosynthetic oxygen production in *Zygogonium* AUT-g and *Zygogonium* SCOT-g is also reflected by the gross photosynthesis:respiration (P:R) ratios, which were lower compared to the respective ‘purple morphs’ at temperature steps with positive net photosynthesis (AUT: 5°C–30°C; SCOT: 5°C–35°C; Fig. 7B). Both Austrian morphs showed the highest P:R ratios at 5°C, while in both Scottish morphs the highest P:R ratios values occurred at 10°C (Fig. 7B).

### Optimum quantum yield of PSII (F_v_/F_m_)—desiccation response

**Figure 8.**
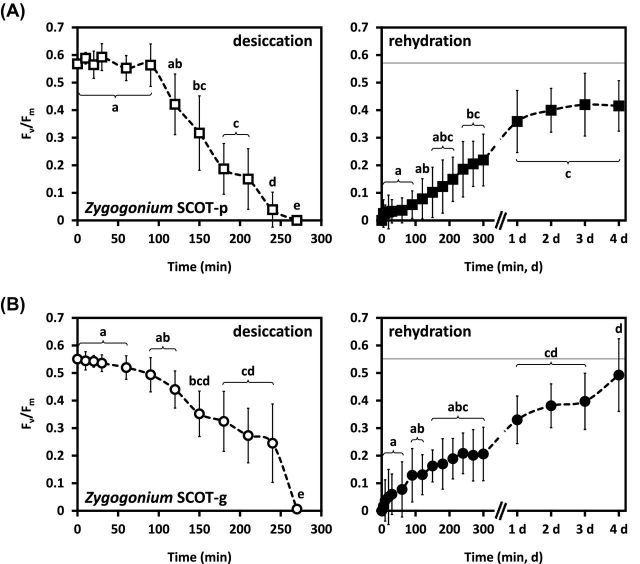
Effect of desiccation followed by subsequent rehydration on the maximum quantum yield of PSII (F_v_/F_m_; *n* = 6 ± SD) of *Zygogonium* SCOT-p (**A**) and *Zygogonium* SCOT-g (**B**). The control Fv/Fm value is indicated as a dashed grey line on the rehydration charts (right). Significant different effects of desiccation and rehydration on the F_v_/F_m_ values over time (small letters) were determined by one-way ANOVA followed by Tukey's post hoc test (*P* < 0.05).

In *Zygogonium* SCOT-p, desiccation at ambient air reduced the initial F_v_/F_m_ value (0.57 ± 0.02) linearly after 90 min (*P* < 0.05), reaching 0 after 270 min (Fig. 8A). Subsequent rehydration with culture medium started recovery of F_v_/F_m_ after 5 min., while after 300 min ∼40% of the initial value was restored (Fig. 8A). After 1 day of recovery, F_v_/F_m_ increased to ∼60% of the initial value and remained unchanged (*P* < 0.05) for 4 days (Fig. 8A). In *Zygogonium* SCOT-g, the initial F_v_/F_m_ value (0.55 ± 0.05) started decreasing significantly (*P* < 0.05) after 60 min of desiccation and reached ∼45% of the initial value after 240 min (Fig. 8B). After 270 min, F_v_/F_m_ reached 0 (Fig. 8B). Rehydration started recovery of F_v_/F_m_ immediately and after 300 min, ∼30% were restored (Fig. 8B). F_v_/F_m_ increased to ∼60% after 1 day, followed by another significant increase (*P* < 0.05) to ∼90% of the initial F_v_/F_m_ value after 4 days (Fig. 8B).

### Microscopic imaging PAM

**Figure 9.**
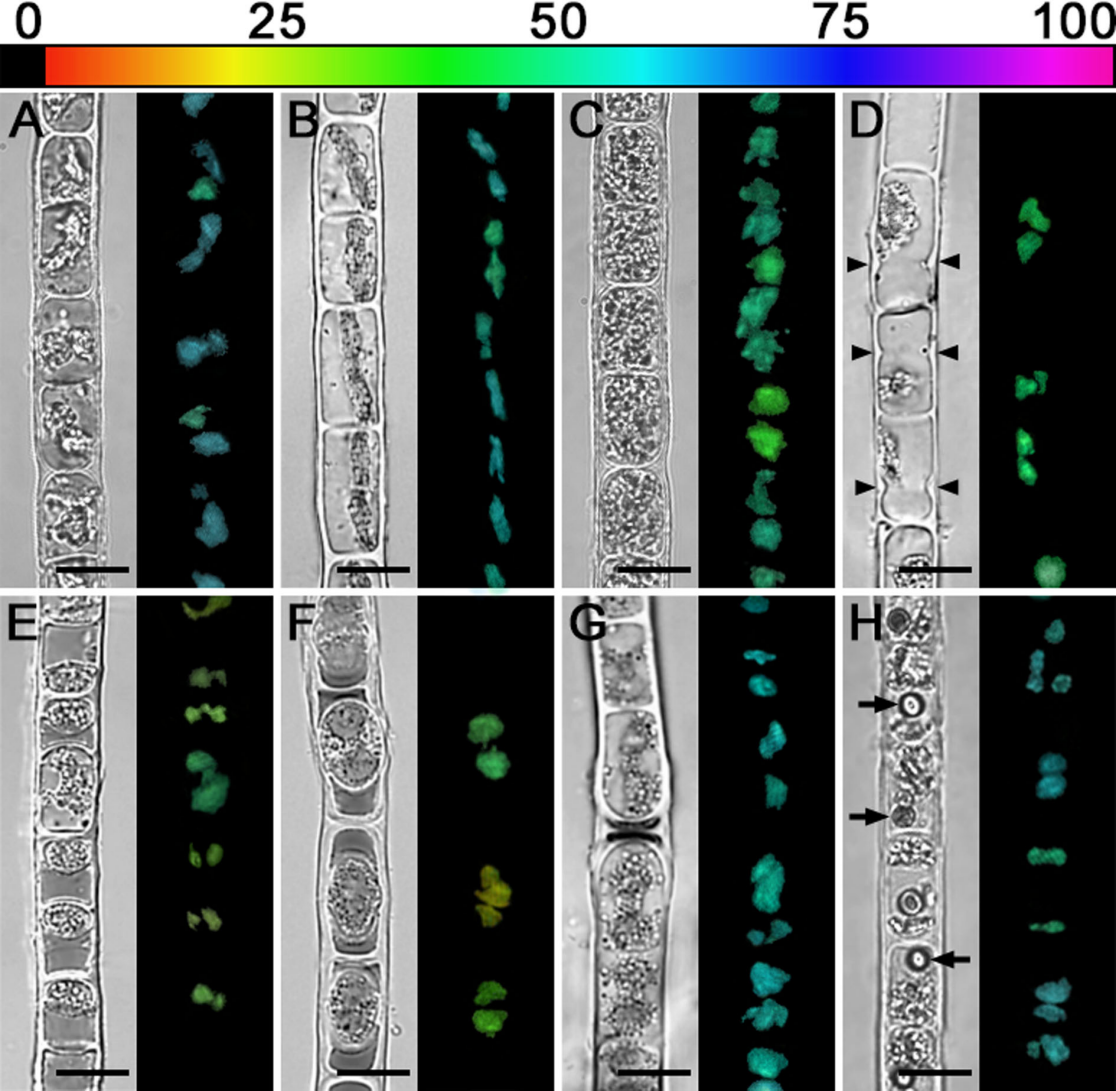
NIR and corresponding Y(II) images (false colour) of *Zygogonium* AUT filaments with different cell morphologies. The relative Y(II) as a percentages is indicated by the colour bar at the top. (**A**) *Zygogonium* AUT-p. (**B**) *Zygogonium* AUT-g. (**C**) Purple filament with older cells. (**D**) Cells depositing cell wall material close to cross cell walls (arrowheads). (**E**) Filament with numerous aplanospores close to cross cell walls of vegetative cells. (**F**) Encysted cells. (**G**) Germinated aplanospores as indicated by dark remnant of cytoplasmic residue. (**H**) Purple filament with one spherical inclusion per cell. Bars = 20 μm.

Using the microscopic version of an Imaging, PAM allowed to visualize the effective quantum yield of PSII [Y(II)] on a cellular level. Most cells of *Zygogonium* AUT-p (Fig. 9A) and *Zygogonium* AUT-g (Fig. 9B) exhibited a similar Y(II) (∼0.61), while only in some chloroplasts the value was lower (Fig. 9A, B). Older filaments (indicated by cells with thick cell walls and filled with storage compounds) showed a lower Y(II) (Fig. 9C). Similarly, cells depositing cell wall material close to the cross cell walls (Fig. 9D), aplanospores (Fig. 9E), or encysted cells (Fig. 9F) showed a Y(II) which amounted to ∼50% of the value measured in usual vegetative cells. Germinated aplanospores and adjacent cells (Fig. 9G) as well as cells containing one conspicuous spherical inclusion (Fig. 9H) showed a similar Y(II) like usual green or purple cells.

## DISCUSSION

The colonization of land by zygnematalean green algae was one of the most pivotal events in the history of life on earth, as it founded the rise of Embryophytes, which shape the extant ecosystems on earth's surface (Waters 2003; Harholt, Moestrup and Ulvskov 2015). These multicellular land plants are characterized by a high level of functional specialization allowing them to partition complementary tasks among different cells, tissues or organs simultaneously (e.g. the mesophyll photosynthesizes, while cutinized epidermal tissues decrease water loss and roots accumulate absorbed heavy metal ions to prevent their harmful spread throughout the plant). In contrast, functional specialization in most algae is restricted to individual cells and the formation of different phenotypes of the same cell lineage (Grosberg and Strathmann 2007). In the vegetative stage, the zygnematalean alga *Z. ericetorum* forms different cell morphologies in response to the environment (Transeau 1951; Stancheva *et al.*^2014^, ^2016^). Therefore, this filamentous alga serves as an ideal model organism to investigate cellular reorganization processes, involving morphologically different cells in the ancestors of Embryophytes.

### Photophysiology of *Zygogonium*

The most striking cell differentiation of *Z. ericetorum* is the formation of purple pigmentation (Lagerheim 1895; Alston 1958; Holzinger, Tschaikner and Remias 2010; Stancheva *et al.*^2014^), which strongly influences the eco-physiological performance. In contrast to the Austrian and Scottish ‘green morphs’ established in the lab (i.e. *Zygogonium* AUT-g and *Zygogonium* SCOT-g), the field collected ‘purple morphs’ (Zygogonium AUT-p, Zygogonium SCOT-p) lacked photoinhibition at 500 μmol photons m^−2^ s^−1^. This corresponds to previous rETR measurements in a purple and green morph of *Z. ericetorum* isolated from Obergurgl (Austria; Aigner *et al.*^2013^). Interestingly, the light response kinetics of the green and purple morphs investigated in the present study were not strongly correlated throughout the applied light range (0–500 μmol photons m^−2^ s^−1^): while both the green and purple morph of *Zygogonium* AUT or *Zygogonium* SCOT, showed similar low light key figures (α and I_c_ value), the light saturation points and P_max_ values were always significantly higher in the ‘purple morphs’. This indicates that the pigment acts as a sunscreen and protector against harmful irradiation. It absorbs highly in the UVR and PAR range (Newsome, Murphy and van Breemen 2013) and gets lost in the absence of UVR (Aigner *et al.*^2013^) or in standard growth medium exhibiting low concentration of iron, which is part of the purple pigment. Aigner *et al.* (2013) stated that *Z. ericetorum* lacks UV-absorbing mycosporine-like amino acids or secondary carotenoids, making phenolic compounds particularity important for photoprotection. Advantageously, phenolics do not contain nitrogen, which allows *Z. ericetorum* to occur in oligotrophic environments such as high alpine streamlets (Obergurgl, Austria) or gravely soils (Glen Coe, Scotland) or even xeric sandy soilcrusts (Hoppert *et al.*^2004^). Only upper layers of *Z. ericetorum* populations synthesize high amounts of the purple ferric (gallate)_2_ complex, which is part of a polysaccharide (MW 30 kDa) of variably linked and branched glucose monomers (Newsome, Murphy and van Breemen 2013). In these sun-exposed filaments, photosynthesis is rarely light limited and allows cells to produce and maintain high concentrations of this carbon-rich pigment by increasing the photosynthetic performance. Interestingly, both the purple and green morph of *Zygogonium* SCOT exhibited a significantly lower photosynthetic oxygen production and consumption compared to *Zygogonium* AUT, while the PI curve kinetics were very similar, pointing to a high phenotypic plasticity (Lürling 2003; Herburger, Karsten and Holzinger 2016). On the other hand, photosynthesis (F_v_/F_m_) in *Zygogonium* SCOT showed a higher resistance against desiccation stress and recovery upon rehydration when compared with *Z. ericetorum* isolated from Obergurgl (Austria; Aigner *et al.*^2013^). Since *Zygogonium* SCOT-p occurred in a gravely soil habitat, where rainwater was the only source of available water, a stimulation of defence mechanisms against cellular water loss via hardening can be assumed (Pichrtová, Hajek and Elster 2014a). Furthermore, measuring temperature dependence of photosynthesis revealed the highest P:R ratio to occur at low temperatures and in the ‘purple morphs’ (*Zygogonium* AUT: 5°C; *Zygogonium* SCOT: 10°C), which reflects the temperature regime in the respective natural habitats (Table S1, Supporting Information). Thus, the upper layers of purple algal filaments, which are exposed to the atmosphere, can gain sufficient amounts of fixed carbon, which is not used for respiration and available for glycosylated pigment and/or biomass synthesis (e.g. formation of aplanospores). This is also supported by the observation that *Zygogonium* SCOT-p exhibited several Golgi bodies close to the chloroplast, the primary location for carbohydrate synthesis in plants.

### Formation of aplanospores removes iron from the protoplast

Using iron in acidic environments as a component of vacuolated sunscreen pigments is not only an effective strategy to cope with harmful solar and UV radiation. It also shifts excessive iron from the cytoplasm and chloroplasts into the vacuoles and reduces the risk of forming reactive oxygen species due to the Fenton reaction. The acidic habitats of *Zygogonium* AUT-p and *Zygogonium* SCOT-p (pH = ∼4.6) exhibited a much higher iron content (∼1.1 mg Fe L^−1^) when compared to more alkaline soil solutions in the region of Obergurgl (pH = ∼6.2–8.6) predominated by a naturally occurring ‘green morph’ of *Z. ericetorum* and other zygnematalean green algae (0.08–0.18 mg Fe L^−1^), Southwest Scotland (0.22 mg Fe L^−1^; Grieve 1990) or usual drinking water (0.30 Fe mg L^−1^; World Health Organization 1996). Excessive concentrations of heavy metals such as iron cause acute and chronic toxic effects on the metabolism of even metal tolerant plants (Nagajyoti, Lee and Sreekanth 2010). In algae, three principal mechanisms allow to maintain non-toxic concentrations of metal ions inside cells (Gaur and Rai 2001): (i) exclusion (e.g. binding to extracellular polysaccharides), (ii) prevention of bioavailability by complexing them inside the cells (e.g. binding to class III metallothioneins; Perales-Vela, Peña-Castro and Canizares-Villanueva 2006) and (iii) excretion from the protoplasts. *Z. ericetorum* cells use all three strategies to cope with iron-rich habitats by (i) binding iron to the extracellular matrix with a maximum in H-shaped cell wall structures between individual cells and by (ii) incorporating iron into a glucose-rich phenolic vacuolar compound. Finally, (iii) aplanospore formation shifts iron-rich compounds to cell poles of vegetative cells; as a consequence, iron is excluded from emerging aplanospores and rendered inert in the extracellular space surrounded by the parental cell wall. These are the areas, where cell detachment occurs frequently, which releases iron-rich residues from the filament into the environment and explains the lower iron content in the ‘green morph’. Microscopic imaging PAM revealed that the effective quantum yield of PSII [Y(II)] of *Zygogonium* AUT-g (low iron content) and *Zygogonium* AUT-p (high iron content) as well as germinated aplanospores was similar (∼0.61). This indicates a fine regulation of iron homeostasis, allowing *Z. ericetorum* to occur in acidic (pH = ∼4.6) iron rich as well as more alkaline habitats (pH = ∼6.2–6.4) with lower iron concentrations, where the formation of aplanospores as a mechanism of active iron exclusion was not observed.

### Aluminium is abundant in the cell wall of the green and purple morph

*Zygogonium* AUT-p contained ∼3.5-fold more aluminium when compared with *Zygogonium* AUT-g as shown by ICP OES. Haematoxylin and morin staining revealed that both morphs enriched this metal in their cell walls, while only the ‘purple morph’ showed morin staining in some chloroplasts with a maximum in the pyrenoid regions. The accumulation of aluminium inside cells of the ‘purple morph’ might be explained by a higher aluminium concentration and a lower pH value (∼4.6) in the soil water when compared with the culture medium (pH = 6.8), where the ‘green morph’ was generated. Aluminium solubility and phytotoxicity are influenced by the pH value of the solution the organisms live in (Godbold, Jentschke and Marschner 1995; Crémazy, Campbell and Fortin 2013). In the streptophyte green algae *Chara corallina*, aluminium transport across the plasma membrane showed a maximum at pH 4.3 and dropped strongly at pH 5.2 (Taylor *et al.*^2000^). The ‘purple morphs’ of *Z. ericetorum* investigated in the present study occurred in habitats with a pH of ∼4.6, where transport of Al^3+^ across membranes is still possible (Taylor *et al.*^2000^), explaining the accumulation in chloroplasts, which was also found in several land plants (Cuenca, Herrera and Merida 1991; De Andrade *et al.*^2011^) but not in the ‘green morphs’ established in the lab. In contrast, aluminium accumulation in the cell wall is less pH depended (Taylor *et al.*^2000^). Like land plants, the cell walls of Zygnematophyceae contain homogalacturonan (Popper *et al.*^2011^), which consists of an α-D-1,4-galacturonan core (Willats *et al.*^2001^). In the cell wall, homogaladcturonan is de-methylesterified by pectin methylesterase enzymes (Domozych *et al.*^2014^), resulting into free carboxylic groups, which are the major binding sites of aluminium ions (Schmohl *et al.*^2000^). Under culture conditions, aluminium accumulation in the cell wall depends on the presence of lipid peroxidizing Fe^2+^, which mediates the production or leakage of new pectins as a primary ligand for aluminium binding (Chang, Yamamoto and Matsumoto 1999). Furthermore, the modulation of the pectin content modifies aluminium sensitivity in plant roots (Schmohl and Horst 2000; Eticha, Stass and Horst 2005), while the amount of de-methylesterified pectins correlates positively with aluminium accumulation and sensitivity due to increased binding of this metal to the more negatively charged cell wall (Horst *et al.*^1999^). Both the ‘purple morph’ of natural field populations of *Z. ericetorum* from Obergurgl (Austria; Holzinger, Tschaikner and Remias 2010) and *Zygogonium* SCOT-p can produce massive layered cell walls with a diameter up to 4 μm. Thus, the drastically higher aluminium content of *Zygogonium* AUT-p might be explained by different cell wall chemistries, namely a higher content of pectins and/or degree of de-methylation (Eticha, Stass and Horst 2005). In general, streptophyte green algae can modify their cell wall composition in response to environmental conditions (Herburger and Holzinger 2015) and increase the pectin content strongly under e.g. desiccation stress (Pichrtová, Kulichová and Holzinger 2014b, and references therein). Binding of Al^3+^ to the pectic matrix decreases the mechanical extensibility of the cell wall and reduces cell growth and development, likely by limiting the movement of wall-loosening enzymes (Eticha, Stass and Horst 2005). Interestingly, aplanospores of *Z. ericetorum* form their own cellulosic wall embedded in the pectin-rich parental cell wall (Stancheva *et al.*^2014^), while aluminium has a very low-binding affinity to cellulose (Chang, Yamamoto and Matsumoto 1999). This *de novo* formation of a cellulosic wall around the spores, followed by their germination inside the filament, allows cell division and filament growth independently of the stiff aluminium cross-linked pectic matrix of the parental wall. Haematoxylin staining in germinated aplanospores of green *Zygogonium* filaments showed that pectic cross-wall protuberances (as shown for *Desmidium swartzii* (Zygnematophyceae); Andosch *et al.*^2015^) and H-shaped cell wall structures between individual cells were particularly rich in aluminium. The latter structures, which are also stained by morin, are remnant of parental cell walls originated from several cell division and wall formation cycles which rupture and detach over time. After transferring the purple field samples of *Zygogonium* AUT-p to aluminium-free culture medium, green cells (*Zygogonium* AUT-g) still contained considerable amounts of aluminium, which was restricted to the cell wall. This might reflect the non-exchangeable fraction of aluminium (Tice, Parker and DeMason 1992), which is tightly bound to the pectic matrix (yet stainable by haematoxylin and morin) with a maximum in distinctly layered H-shaped cell wall pieces still attached to cells and germinated aplanospores. Over time, these pieces detach from the cells due to rupture or mechanical strain. They might be also related to cell detachment (Stancheva *et al.*^2014^; Herburger, Karsten and Holzinger 2016).

### High zinc content does not affect photosynthetic performance

In contrast to aluminium, *Zygogonium* AUT-g contained very low amounts of zinc, while *Zygogonium* AUT-p showed similar values like aluminium. Zinc can decrease growth, cell division rate and the photosynthetic activity of Zygnematophyceae (Volland *et al.*^2011^). Furthermore, uptake of zinc results into strongly enlarged mitochondria with bloated cristae (Andosch *et al.*^2015^), which was also observed in the ‘purple morph’ of *Z.* ericetorum isolated from Obergurgl (Austria; Holzinger, Tschaikner and Remias 2010) and *Zygogonium* SCOT-p. However, negative effects on photosynthesis were not observed, since *Zygogonium* AUT-p (high zinc content) showed similar Y(II) values when compared with *Zygogonium* AUT-g (low zinc content) and even a higher photosynthetic O_2_ production and respiratory consumption. Therefore, similar to *Micrasterias denticulata* (Zygnematophyceae) and several land plants (Volland *et al.*^2011^ and references therein), Zn might be detoxified by compartmentalization in the cell wall, vacuoles and mucilage vesicles, while the latter might be involved in removing excessive Zn from the protoplast.

## CONCLUSION

Based on the observations in the present study, we consider the formation of aplanospores as a rejuvenation process, allowing *Z. ericetorum* to survive in acidic metal-rich habitats, where other zygnematalean algae (*Zygnema* sp., *Spirogyra* sp.) cannot occur: (i) Spore formation shifts excessive iron-rich compounds in the cell periphery and excludes them from emerging unpigmented aplanospores; (ii) spore germination allows filament growth despite high amounts of aluminium, iron and likely zinc in the pectin-rich parental cell wall matrix. Both the purple and green morph of *Z. ericetorum*, maintain a high photosynthetic performance in acidic (pH = ∼4.6) and neutral (pH = 6.8) environments, while the presence of the purple iron-rich pigment coincides with an considerable higher photosynthetic oxygen production in response to higher light intensities up to 500 μmol photons m^−2^ s^−1^ and temperature gradients (5°C–45°C). Different responses to light, temperature and desiccation gradients followed by rehydration in *Zygogonium* AUT and *Zygogonum* SCOT imply a high phenotypic plasticity and capability to respond to different limnetic transition habitats, which we consider to be crucial for the evolutionary landmark terrestrialization.

## Supplementary Material

Supplementary Data
