# Supplementary material for: The green alga Zygogonium ericetorum (Zygnematophyceae, Charophyta) shows high iron and aluminium tolerance: protection mechanisms and photosynthetic performance
Source: FEMS Microbiol Ecol. 2016 May 12;92(8):fiw103. doi: 10.1093/femsec/fiw103 (PMC4909054; doi:10.1093/femsec/fiw103)
Supplement: Supplementary Data [file fiw103_supplementary_data.zip › Supplementary_Table_S1.docx]

Supplementary Table S1. Origin of *Zygogonium* AUT-p and *Zygogonium* SCOT-p.

| **Isolate** | **Habitat** | **Metrological data** |
| --- | --- | --- |
| *Zygogonium ericetorum* AUT ‘purple morph’  (*Zygogonium* AUT-p) | Streamlet on Mt. Schönwieskopf (~2300 m a.s.l.), Tyrol, Austria (46°50'52.9"N 11°00'55.4"E); isolated on 18 August 2013; water depth 5-20 cm, open water surface; air temperature: 8.5 °C | Min. air temperature: -8-6 °C  Max. air temperature: -3-12 °C  Monthly rainfall days: 11-18  Monthly precipitation: 36.3-120.3 mm  Annual rainfall: 839 mm |
| *Zygogonium ericetorum* SCOT ‘purple morph’  (*Zygogonium* SCOT-p) | Wayside near Glencoe (~45 m a.s.l.), Lochaber, Scotland (56°41'05.6"N 5°05'25.9"W); isolated on 16 Aug 2014;  algal filaments covered by thin water layer; air temperature: 16.9 °C | Min. air temperature: -0.7-9.7 °C  Max. air temperature: 5.5-17.4 °C  Monthly rainfall days: 14-20  Monthly precipitation: 77.0-248.3 mm  Annual rainfall: 1809.4 mm |

Habitat characteristics and meteorological data (temperatures expressed as means; www.worldweatheronline.com, http://www.metoffice.gov.uk) are given.
