# Supplementary figures and images for: The green alga Zygogonium ericetorum (Zygnematophyceae, Charophyta) shows high iron and aluminium tolerance: protection mechanisms and photosynthetic performance
Source: FEMS Microbiol Ecol. 2016 May 12;92(8):fiw103. doi: 10.1093/femsec/fiw103 (PMC4909054; doi:10.1093/femsec/fiw103)

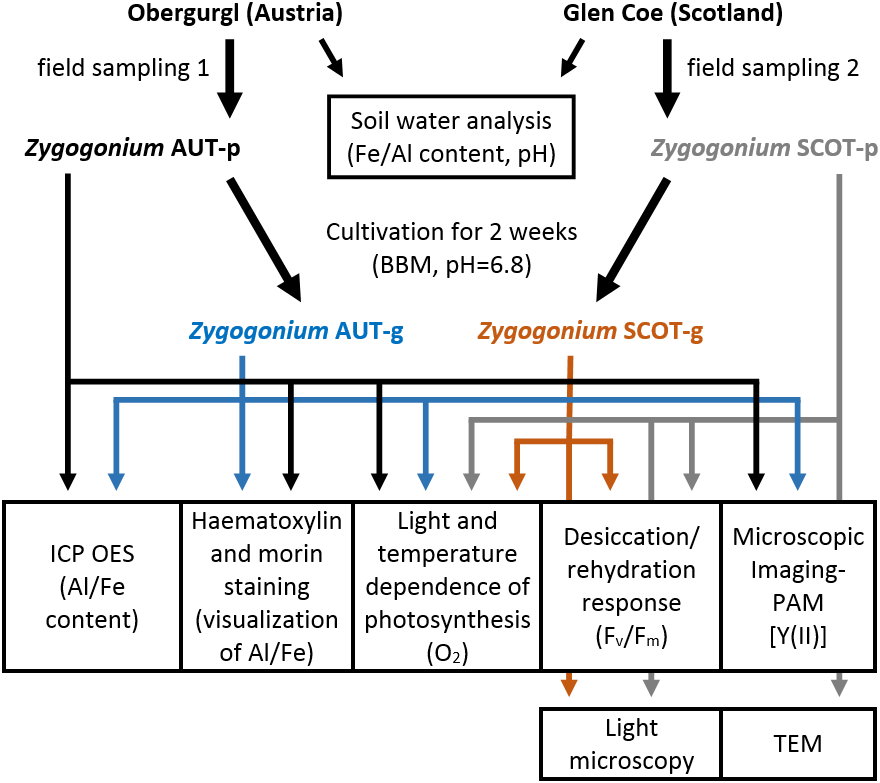

Supplement: Supplementary Data [file fiw103_supplementary_data.zip › Supplementary_Fig_S1.tiff]

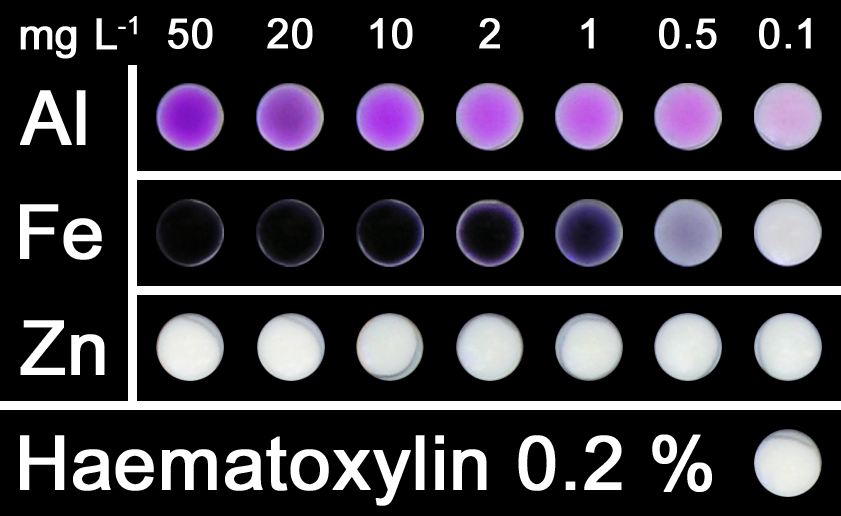

Supplement: Supplementary Data [file fiw103_supplementary_data.zip › Supplementary_Fig_S2.tiff]

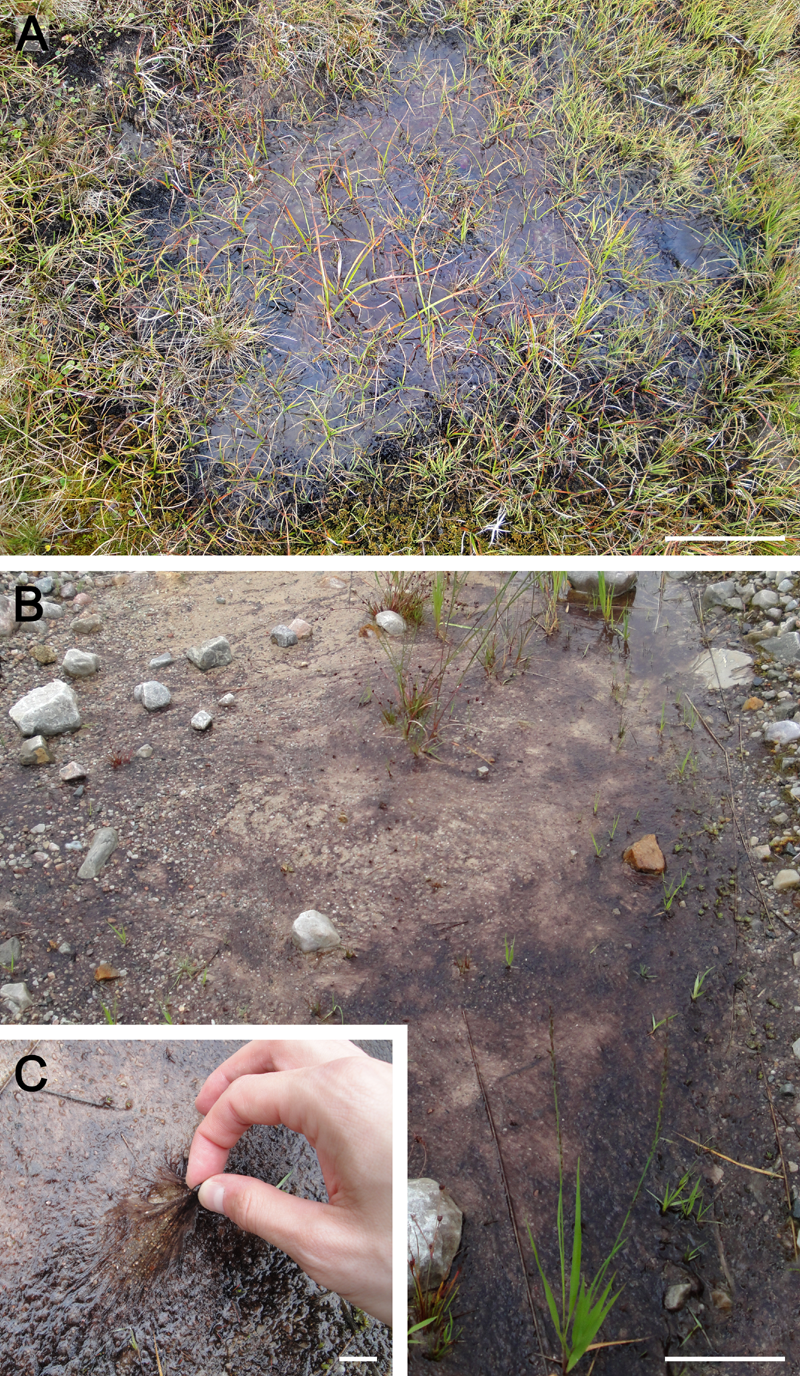

Supplement: Supplementary Data [file fiw103_supplementary_data.zip › Supplementary_Fig_S3.tiff]
