# Supplementary material for: The green alga Zygogonium ericetorum (Zygnematophyceae, Charophyta) shows high iron and aluminium tolerance: protection mechanisms and photosynthetic performance
Source: FEMS Microbiol Ecol. 2016 May 12;92(8):fiw103. doi: 10.1093/femsec/fiw103 (PMC4909054; doi:10.1093/femsec/fiw103)
Supplement: Supplementary Data [file fiw103_supplementary_data.zip › Supplementary Figure Legend.doc]

Supplementary Fig. S1

Schematic overview on the methods applied to the four different algal samples (*Zygogonium* AUT-p, *Zygogonium* AUT-g, *Zygogonium* SCOT-p, *Zygogonium* SCOT-g) originating from Obergurgl (Austria) or Glen Coe (Scotland).

Supplementary Fig. S2

Aqueous solutions of haematoxylin are used to identify iron or aluminium deposits in cells and tissues (Culling et al. 1985), while iron tends to form black and aluminium lilac to violet haematoxylin complexes (Arroyave et al. 2013; Hajiboland et al. 2013). However, aluminium treated root tips appeared black after haematoxylin staining (De Andrade et al. 1997; Doncheva et al. 2005). Testing of the haematoxylin solution used in the present study: 25 µL droplets of AlCl_3_ (Al), FeCl_3_ (Fe) and ZnCl_2_ (Zn) solutions (pH = 4.6) with decreasing concentrations (50–0.1 mg metal L^-1^) stained with 0.2 % haematoxylin for 45 min. Al: lilac to pale lilac, Fe: blackish to colorless, Zn: colorless, haematoxylin 0.2 %: colorless.

Supplementary Fig. S3

Habitat of *Zygogonium* AUT-p (A) and *Zygogonium* SCOT-p (B, C). (A) Shallow pond containing abundant submersed purple algal filaments. (B) Gravelly soil covered by purple algal filaments with a maximum in a rainwater filled draw (right). (C) Thin layers of filaments are easy separable from the soil.

Supplementary References

Arroyave C, Tolrà R, Thuy T, et al. Differential aluminum resistance in *Brachiaria* species. Environ Exp Bot 2013;89:11–18.

Culling CFA, Allison RT, Barr WT. Cellular pathology technique. 4th ed.: London, Boston: Butterworthsin, 1985.

Doncheva S, Amenós M, Poschenrieder C, et al. Root cell patterning: a primary target for aluminium toxicity in maize. J Exp Bot 2005;56:1213-1220.

Hajiboland R, Barceló J, Poschenrieder C, et al. Amelioration of iron toxicity: A mechanism for aluminum-induced growth stimulation in tea plants. J Inorg Biochem 2013;128:183-187.

De Andrade LRM, Ikeda M, Ishizuka J. Localization of aluminum in root tip tissues of wheat varieties differing in aluminium tolerance. J Fac Agr Kyushu Univ 1997;41:151-156.
